# A Unified Efficient Deep Learning Architecture for Rapid Safety Objects Classification Using Normalized Quantization-Aware Learning

**DOI:** 10.3390/s23218982

**Published:** 2023-11-05

**Authors:** Okeke Stephen, Minh Nguyen

**Affiliations:** Computer Science & Software Engineering, Auckland University of Technology, Auckland 1010, New Zealand; minh.nguyen@aut.ac.nz

**Keywords:** deep learning ensemble, rapid object classification, onsite personnel identification, normalized quantization-aware learning, complex industrial scene

## Abstract

The efficient recognition and classification of personal protective equipment are essential for ensuring the safety of personnel in complex industrial settings. Using the existing methods, manually performing macro-level classification and identification of personnel in intricate spheres is tedious, time-consuming, and inefficient. The availability of several artificial intelligence models in recent times presents a new paradigm shift in object classification and tracking in complex settings. In this study, several compact and efficient deep learning model architectures are explored, and a new efficient model is constructed by fusing the learning capabilities of the individual, efficient models for better object feature learning and optimal inferencing. The proposed model ensures rapid identification of personnel in complex working environments for appropriate safety measures. The new model construct follows the contributory learning theory whereby each fussed model brings its learned features that are then combined to obtain a more accurate and rapid model using normalized quantization-aware learning. The major contribution of the work is the introduction of a normalized quantization-aware learning strategy to fuse the features learned by each of the contributing models. During the investigation, a separable convolutional driven model was constructed as a base model, and then the various efficient architectures were combined for the rapid identification and classification of the various hardhat classes used in complex industrial settings. A remarkable rapid classification and accuracy were recorded with the new resultant model.

## 1. Introduction

**Figure 1:**
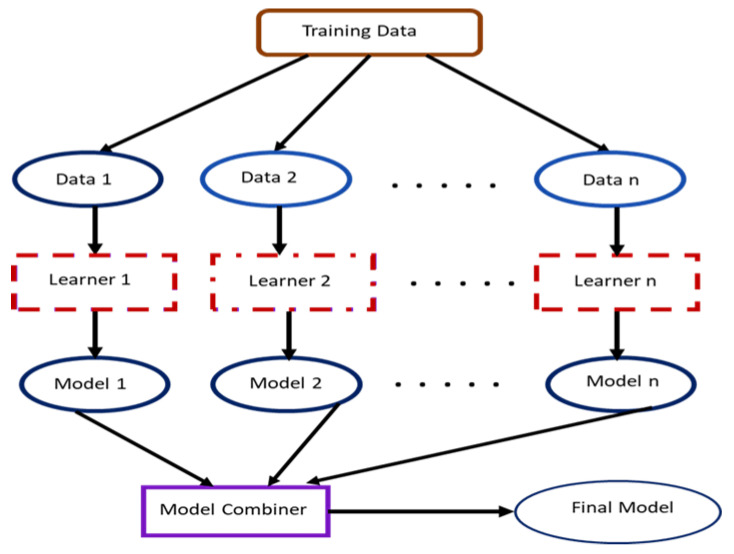
A cross-section of a unified model framework.

The concept of contributory learning, otherwise generally referred to as ensemble learning, merges different pieces of model architectures (see Figure 1) to build a unified model that offers superior learning features and generalization for enhanced object classification performance. Deep artificial learning models that possess diverse processing architecture provide a more outstanding performance against conventional or shallow object classification models. By extension, the unified contributory learning models blend the advantages of the artificial deep learning models to derive a final model with an improved generalization performance.

The object classification task deals with new observation categorization, relying on a hypothesis derived or learned from a collection of training data. The mapping of the features of input data to their corresponding or fitting labels represents the hypothesis that the core objective is to relatively approximate the actual undetermined function as closely as possible to minimize generalization errors [1]. Despite the efforts of the single architectures to reduce the generalization errors, it is challenging to attain a satisfactory performance, especially with inadequate, unbalanced, noisy, and high-dimensional complex data [2]. This is orchestrated by the single models’ difficulty in capturing the multiple features embedded in the input data and their corresponding structures. It, therefore, becomes imperative to construct a rapid, efficient model that can learn the various characteristics of complex data efficiently.

In the unified learning concept, a collection of features that passes through diverse transformations is initially extracted and learned. Then, multiple learning models or algorithms are deployed to generate weak predictive results based on the learned features before the fusion of the individually learned features by an intermediate mechanism to produce a superior discriminative or classification framework. A conventional unified learning model consists of two phases: (1) the initial classification result production step with various weak classifiers and (2) the fusion of the multiple results to form a reliable model to produce an outcome with an appropriate mechanism. There are several extensively deployed ensemble classification schemes in task classifications, such as random forest [3], AdaBoost [4], gradient boosting [5], random subspace [6], etc. Through a training dataset random sampling, the Bagging approach creates sample subsets, which are used to train basic models for inferencing [7].

In random subspace utility, a set of feature subspaces are constructed by performing features random sampling and training basic classifiers in their subspace domains to produce multiple results that are then unified into a single final result [6]. In Gradient Boosting, data are randomly sampled to create a sum of the last residuals by integrating tiny models that forcefully make predictions near the actual value [8]. The ensemble stacking approach is vital in constructing unified, efficient learning models. This method combines the outputs of different base models using an effective selection mechanism to yield a superior classification or predictive model.

The stacking approach deploys a meta-learning concept in task processing to fuse or integrate base models’ outputs [9]. A model blending concept is birthed when a linear model constitutes the last decision-making portion of the stacking model. In the stacking process or stacked regression, the dataset is divided randomly into *D* equal parts. Given a *D^th^* fold cross-validation of the dataset, a set is reserved for the proposed model test while the remaining are used for the model training. The predictions of the various base learning models are obtained using the train test pair subsets of the dataset, which then serve as the meta-data deployed to construct a meta-model. These shallow algorithms lack robustness for learning similar fine-grained features and thus significantly impact the performance of the trained model. This investigation introduces a quantization-aware meta-learning feature-based deep learning architecture fusing method. This method learns the object features, is lightweight, and rapidly processes new objects due to quantization.

## 2. Related Works

In recent years, pieces of literature on deep-learning architectural ensembles have emerged from which our model design concept is derived. In their work, Koitka and Friedrich [9] introduced an ensembled network based on the optimized deep convolutional artificial network for image classifications. They adopted several convolutional neural network architectures similar to our proposed model. During the training process, pre-trained models were fine-tuned and partitioned into two different optimal steps by initially training the logit layers for adaptation to random initialization for the free flow of information and domain dataset. Then, the entire layers are trained by deploying a polynomial decay optimizer.

Also, a neural ensemble-based detection of patterns was proposed to inspect specimens using a two-level deep model architecture ensemble [10]. The first-level ensemble was used to verify a normal cell with a superior confidence score and two expected outputs from each network. The second-level ensemble handled defect cells emanating from the first-level ensembled network. Then, the individual network predictions were fused using the full voting ensemble technique [11]. A weighted convolutional neural network ensemble method [12] was proposed to unify convolutional operation probabilities’ outcomes efficiently. In their work, Nguyen and Pernkopf [13] introduced a CNN-based ensemble technique in conjunction with the nearest neighbor filter to classify acoustic scenes. In the work, they adopted several CNN model architectures for performing single-input and multi-input channel learning and three base models for the ensemble network construct.

In another related investigation, Fawaz et al. [14] ensembled 60 deep-learning models to perform time series classification tasks. Furthermore, joint training for neural network ensembles that use a single loss function to train multiple deep learning architectures with multiple branches was investigated [15]. The study introduced a collection of novel loss functions that generalized several different previous techniques, and their theoretical and empirical characteristics were thoroughly examined for joint training tasks. The presented method in this work adheres to the aforementioned principles and practices with an improved network fusing layer.

Furthermore, a rapid tropical cyclones (TCs) intensification (RI) predictor prosed for safety [16] and a production forecasting of coalbed methane using a DL-driven ensemble learning method for complex production patterns [17]. In other studies, Li and Hong [18] deployed the DL ensemble learning method to model flood susceptibility for the reduction of loss orchestrated by flooding. Yazdinejad et al. [19] introduced a cyber threat-hunting model that utilizes ensemble deep learning in the industrial internet of things (IIoT) platforms. Four-dimensional modeling in an industrial context typically refers to the use of 3D modeling and visualization technology to incorporate the dimension of time, creating a comprehensive representation of a project’s evolution over time. Kyriakaki et al. [20] investigated a 4D reconstruction of tangible cultural heritage objects from web-retrieved images and a review of the incorporation of laser scanning and photogrammetry in 3D/4D cultural heritage preservation and the application areas of 3D and 4D models on construction projects was completed [21,22].

### Contributions

Our research paper makes several significant contributions to the field of safety object classification and deep learning:

1. Unified Architecture: We introduce a unified deep learning architecture that is specifically designed for safety object classification, offering a comprehensive solution to the challenges associated with real-time safety systems.
2. Normalized Quantization-Aware Learning: We propose and validate the effectiveness of Normalized Quantization-Aware Learning, a novel approach that combines quantization and normalization techniques to improve both the speed and accuracy of safety object classification.
3. Experimental Evaluation: We provide a comprehensive evaluation of our architecture through extensive experiments, showcasing its superior performance in terms of accuracy, speed, and memory efficiency when compared to existing methods.
4. Real-world Applicability: We emphasize the practicality of our approach by demonstrating its effectiveness in real-world scenarios, thereby highlighting its potential for integration into safety-critical applications.

## 3. Theoretical Background

The proposed model comprises a combination of four efficient, lightweight deep learning model architectures, with each model contributing to the unified single-model learning process. The benchmark model for which the test result is used as a yardstick to measure the performance of the proposed model is constructed with separable convolutional neural network layers. The separable convolutional neural network [23] consists of layers split into sub-processes or multiple convolutions to generate the same output during the convolution process. The depth-wise convolution deploys a single convolutional filter for an individual input channel while creating a linear combination of the resultant of the depth-wise convolution process using the pointwise convolution mechanism.

**Figure 2:**
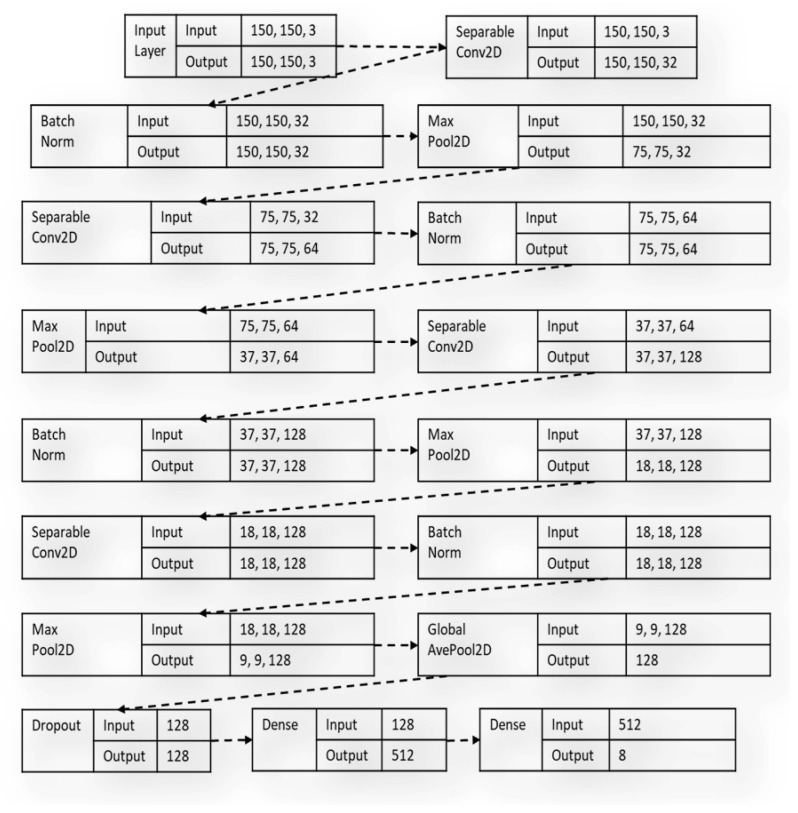
The architectural flow of the benchmark custom model with SeparableConv2D layers.

On the other hand, the spatially separable convolutions decompose convolution operations into two individual processes. In a typical convolution operation [24,25], if a 3 × 3 kernel filter is employed, a sample image can be convolved directly with the kernel. However, in spatially separable convolution, a 3 × 1 kernel is first used to convolve over the given image sample before a 1 × 3 kernel, which is more parameter efficient compared to the conventional convolution layers because of minimal matrix computations. The diagram in Figure 2 shows the benchmark custom model built with the SeparableConv2D layers during the study.

The proposed architecture’s first base model (BM1) is derived from the Inception-V3 architecture. The Inception-V3 [26] is a CNN architecture that is among the Inception model group of networks that enhances the object classification tasks through the incorporation of several techniques such as convolution factorization, regularization, label smoothing, parallelized computations, dimension reduction, and integration of an auxiliary classifier for object label information propagation down the bottom of the network. The convolution factorization assists the model in minimizing the computational complexity of the architecture through network parameter reduction, thereby boosting the network efficiency. The auxiliary classifier is a tiny CNN layer integrated in between the network layers during the training process, and the loss emanating from it is summed into the primary network loss.

The second base model (BM2) is derived from the Xception deep learning architecture [23], inspired by the Inception deep CNN modules. The Xception module convolution layers were swapped with depth-wise separable convolutions, which yields slightly superior performance than the Inception V3 on large, benchmarked image classification datasets. Two notable changes in the Xception architecture exist: the reordering of convolution operations and the integration or non-integration non-linearity function. In the enhanced module, a 1×1 convolution operation is first performed by the depth-wise separable convolution layers before conducting a channel-wise spatial convolution. On the second change, an intermediary ReLU non-linearity function is absent, unlike the typical Inception module incorporated with a non-linearity function after the initial operation.

Furthermore, the third base model (BM3) is made of the DenseNet121 CNN model that uses dense connections between CNN layers via dense blocks in which all layers in the network are directly linked. In the network, the preservation of the working principle of the feed-forward layers is achieved by fusing each layer in the network with extra inputs from prior layers, which then transfer their feature maps to all successive layers. Finally, the fourth base model (BM4) is derived from the MobileNet CNN architecture [27], which also uses depth-wise separable convolutions to construct lightweight artificial deep networks. In the network architecture, two straightforward global hyperparameter strategies were introduced to mitigate and balance the difference between latency and accuracy efficiently. 

Stacking is an ensemble strategy consisting of a two-layer structure for combining the outputs of multiple base architectures through the meta-model learning concept to identify an optimal classification or regression model [27]. The feature learning capabilities of different CNN models can be integrated to form an optimal model for a superior classification model without necessarily increasing the depth of the new networks. This strategy can correct errors emanating from the base models to enhance the performance of the ensembled model by maximizing the learning capabilities of the different contributing models.

In this work, we fuse the features from these efficient architectures using a normalized low-bit precision quantization-aware learning paradigm to arrive at a more efficient and rapid model for the object classification task. The low-bit precision quantization-aware learning reduces the computational and memory requirements of the introduced network by representing the model weight and activations using a lower number of bits. It also helped mitigate the performance degradation due to the quantization process when mapping continuous values to a finite set of discrete values. The quantization process is represented as:

Q(x) = ⌊(x/Δ) ⋅ Δ⌉

where:

Q(x) is the quantized value of x, and Δ is the size of the quantization step, determined by the number of bits used for the quantization process. The quantized weight error is computed using:

E = w − q(w)

where:

E represents the quantization error, w is the model’s original floating-point weight, and q(w) is the quantized weight. The quantized activation error is represented by:

E = a − q(a)

where:

E is the quantization error, a is the original floating-point activation, and q(a) is the quantized activation.

## 4. Material and Method

The experimental procedure and the proposed model evaluation matrices are presented in this section. The experiment was conducted on a high-end GeForce RTX TITAN Xp GPU computer with 12 GB graphic capability, cuDNN, and CUDA Toolkits. The model was trained, validated, and tested in five hours. The model was built on the TensorFlow open-source deep learning framework and Python programming language. The hardhat dataset used for the experiment was curated using camera sensors in different positional angles, and others were scrapped from the web with 1705 samples of eight different classes. The dataset was split into training and test sets with 1225 and 480 samples, respectively. The training set was further divided to obtain the validation set with an 80% and 20% sample split strategy. 

According to the global hardhats color coding system, white identifies the managing team, engineers, supervisors, and forepersons in the construction and industrial settings. This class was labeled as 4 and denoted with “Mg” during the experiments. Furthermore, the blue hardhat color coding identified the electricians and high machine operators and was designated with ET&O and labeled class 0; the pink hardhat color identified the female workers and was represented with “FF” and labeled as class 1 during the experiment. The red color coding recognized the firefighters and was defined with “FW” and marked with class 2. The yellow color coding recognized the laborer and heavy-duty machine operators and was denoted with “LH&C” and classed as 3. In continuation, the safety officers were identified with the green color code, marked with “SO”, and classed with the number 5. Also, the site visitors were coded with gray, denoted as “SV”, and classed with the number 7. Finally, the brown hardhat color coding identified the welders and high-heat equipment operators and was represented with “W&HHO” with the number 7 class.

**Figure 3:**
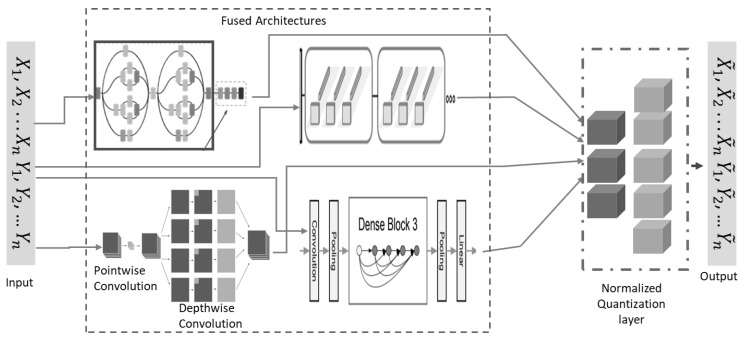
The proposed unified deep-learning architecture.

The hardhats were macro-grouped into eight classes. Due to the high volume of data required for training and testing the reliable deep learning model, data augmentation was used to artificially increase the dataset volume and boost the classification accuracy of the proposed model. The dataset was rescaled to 1/255, 40° rotation range, width shift range of 0.2, height shift range of 0.2, shear range of 0.2, zoom range of 0.2, and horizontally flipped. In the proposed model-building process, a custom benchmark model was first built and trained using five SeparableConv2D layers, as shown in Figure 2. The results of the custom model were used to measure the hardhat classification capabilities of the main proposed architecture. Then, the BM1, BM2, BM3, and BM4 were trained separately and then linearly combined using normalized low-bit precision quantization-aware learning. Each selected architecture and the final proposed model were trained using a learning rate scheduler of 9 × 10^−4^ and compiled using a categorical cross-entropy as a loss function with an Adam optimizer, all trained with 100 epochs. As illustrated in Figure 3, a quantized layer was used to perform the final classification task using a softmax function after the unifying mechanism.

## 5. Results

This section presents a detailed analysis of the proposed method to ensure that the results obtained are interpreted correctly with their corresponding significant relationships spotted. The statistical tests are vital in evaluating the statistical differences in performance of each constructed custom model, the adopted and the proposed models. We measured the accuracy, precision, recall, and F1 scores of all the models involved in the experiment. Also, empirical processes were performed to establish the presence or absence of considerable variations using the performance metrics’ mean values of the custom, individual contributing, and proposed models. Furthermore, the mean square errors (MSE), means-square-log-errors (MSLE), and the Matthews correlation coefficient (MCC) scores were obtained for all the models to reaffirm the proposed model’s performance.

**Figure 4:**
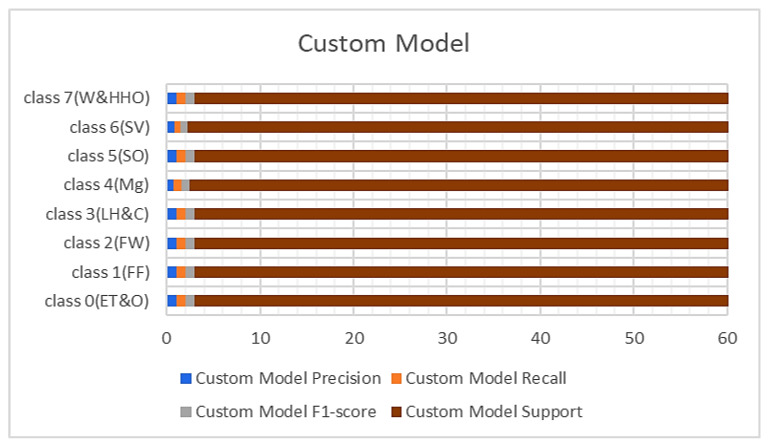
Results from the custom.

**Figure 5:**
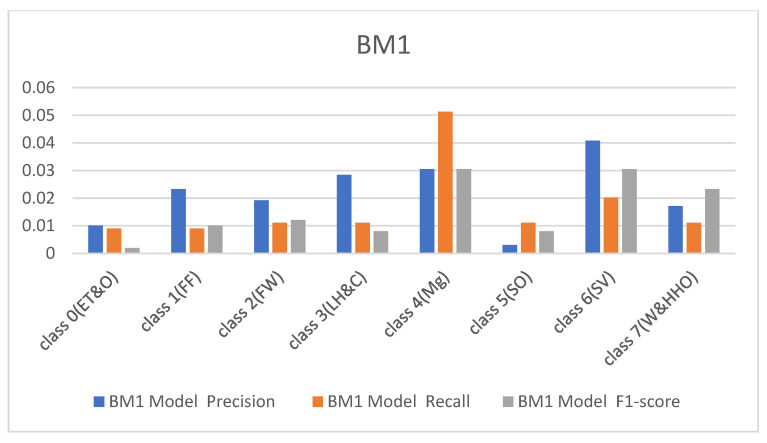
Results from the FBM models.

The performance metrics obtained from the custom-built model and BM1 are shown in Figure 4 and Figure 5 with respect to the precision, recall, and F1 scores. It was observed that the custom model rightly classified all the hardhats from the test data except class 4(Mg), which represents the managers, and class 6(SV), which recognizes the site visitors. The difficulty in classifying these two samples of the hardhat is due to the close relationship between their color coding, i.e., gray and white. The custom model rightly classified 54 samples of the managers’ hardhat and misclassified six samples out of the 60 test samples as site visitors. On the other hand, it appropriately classified 39 samples of the site visitor’s hardhat test samples and wrongly classified 21 samples as the managers’ hardhat (see Table 1). The BM1 also rightly classified all the test samples except the two highly similar classes. It recorded a precision of 87%, recall of 88%, and F1-score of 88% for class 4(Mg) and 88%, 87%, and 87%, respectively, for the site visitor class. This implies that BM1 rightly classified 53 samples as managers and misclassed seven samples as site visitors while rightfully classifying 52 samples as site visitors and wrongly classifying eight samples as managers.

**Figure 6:**
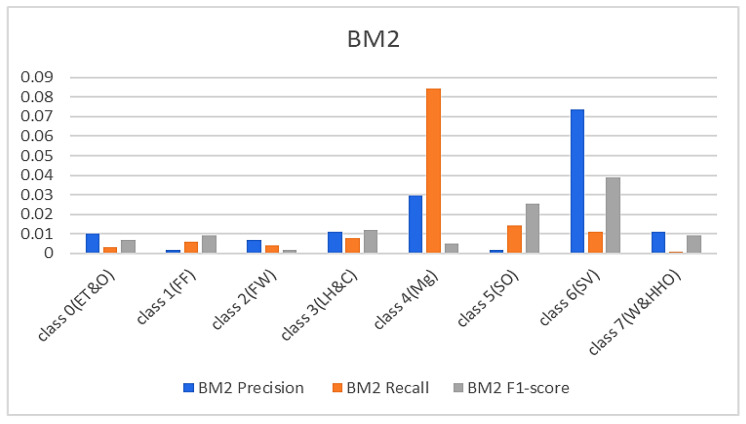
Results from the SBM.

**Figure 7:**
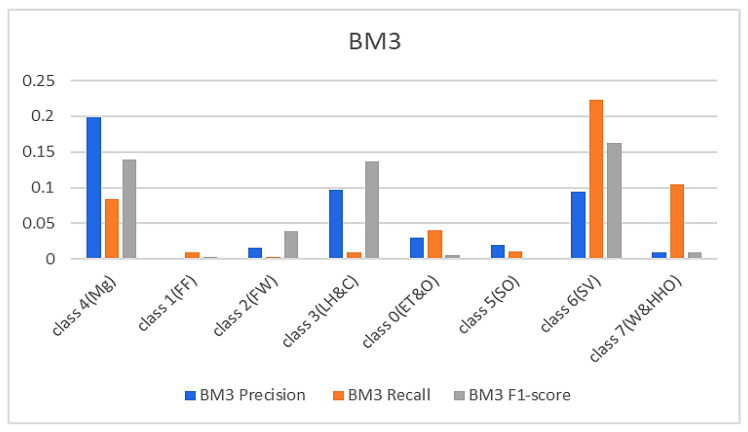
Results from the TBM models.

**Figure 8:**
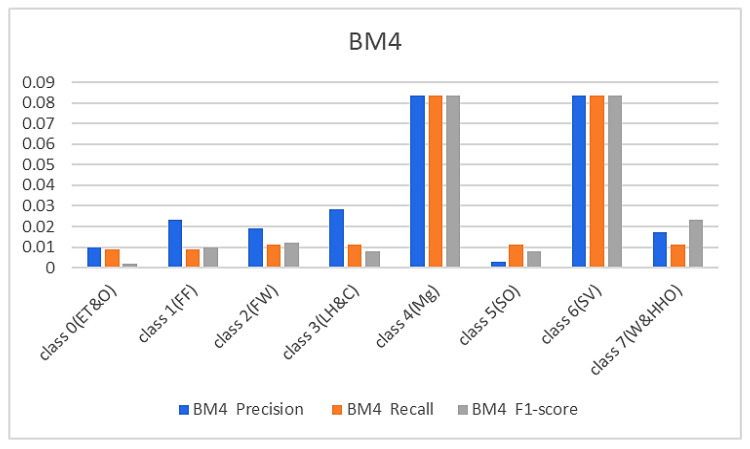
Results from the LBM.

**Figure 9:**
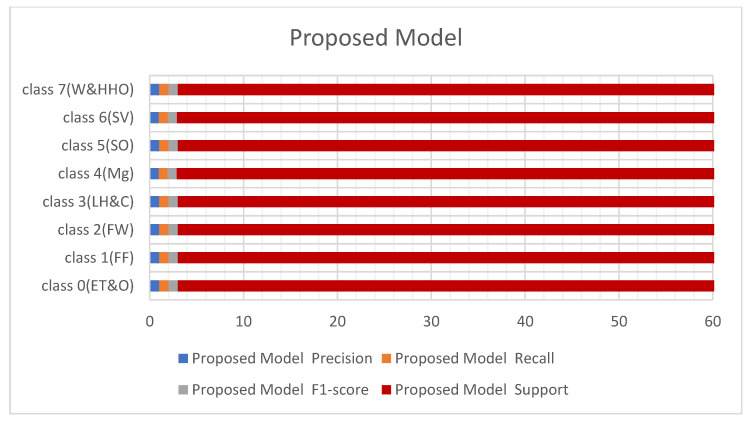
Results from the proposed models.

In continuation, all attention and analysis are focused on the two similar samples since all the models rightly classified all the other test samples except these two. To have a balanced representation of the values extracted from the experiments in charts presented in Figure 6, Figure 7, Figure 8 and Figure 9, the absolute natural logarithm (Ln) of the values was computed and used to plot the various charts. For BM2, a 90% precision, 91% recall, and a 91% F1-score for class 4(Mg) were obtained, and 92%, 90%, and 91% for the site visitor class (see Figure 6 and Figure 7). This indicates that BM2 rightfully classified 55 samples out of the 60 test samples as managers and incorrectly classified five samples as site visitors. The model also rightly classified 54 samples of site visitors and misclassified six samples as managers (see Table 1). The BM3, on the other hand, yielded a 91% precision, 80% recall, and 85% F1 score for class 4(Mg) and 92%, 92%, and 92%, respectively, for the site visitor class. This indicates that BM3 classified 55 samples appropriately as managers and misclassified five samples as site visitors, while 51 were rightfully classified as site visitors and nine were misclassified as managers.

Figure 8 and Figure 9 below represent the results of various metrics using the BM4 and the proposed model. The BM4 produced a 92% precision, recall, and F1-score for class 4(Mg) and the same scores for the site visitor class. This indicates that BM4 rightfully classified 55 samples from managers and site visitors and misclassified five samples each from both classes. On the other hand, the proposed model produced 97% precision, 95% recall, and a 97% F1-score for class 4(Mg) and 96% precision, 98% recall, and a 97% F1-score for class 6(SV), indicating a superior performance compared to the custom-built model and the individual contributing learning models. The proposed model misclassified only three managers’ samples and two site visitors’ samples (see Figure 9).

During the experiments, the test accuracy and mean scores of the various studied models were also observed (see Table 2). The accuracy of the custom model and the adopted co-learner models ranges from 94% to 98%, with the custom-built model yielding the least accuracy, followed by the BM3, while BM2 and BM4 recorded the highest accuracy among the adopted models. The proposed model produced an exceptional accuracy of 99% better than the custom model and the other co-learner models. The custom benchmark model produced a means square error score of 0.23, a mean square log error of 0.006, and an MCC score of 0.94 compared to the introduced model, which produced a lower mean square error of 0.04, MSLE of 0.001, and MCC of 0.99.

With a negligible performance loss, our quantized model reduced significantly from 61 M parameter weight to 4 M parameter weight and processing speed from 45 s to 0.01 s. The deep quantization targets extremely latency-driven applications that run on embedded devices. These devices require real-time inferencing, and our proposed method yields the needed characteristics to function in real-time. 

## 6. Discussion and Conclusions

For the smooth convergence of the constructed custom model, the various co-learning and contributing models, and the actual proposed model, strategies such as hyperparameter optimization, batch normalization through implicit regularizations, learning rate annealing, and aggressive dropouts for enhanced generalization process were judiciously deployed in the various architectures used during the experiments. Table 2 above shows the performance representation of the benchmarked custom, custom, and proposed models. The introduced model outperformed the custom model by gains in convergence and accuracy from 0.94 to 0.99, which is highly significant in the object recognition and classification domain.

Deep CNN architectures are affected by high variance during training and inferencing due to their specific training data dependence. Thus, they are highly prone to overfitting, which causes increased bias and generalization reduction. We checkmated this issue by training different efficient models and obtaining a collection of diverse predictive models. These were then unified to form a single efficient model capable of rapid and optimal object classification. Various base-learning models were selected and experimented with during the experimentation process to determine the best models for rapid and accurate object feature learning. The models were then evaluated for efficiency, accuracy, and the best combination to yield the proposed model. The results of the experiments were statistically evaluated to determine the significant improvement in the trained models.

We also compared the proposed rapid and efficient model with other related models in the literature. Our model yielded superior performance in terms of accuracy, efficiency, and less complex architecture for the classification and identification of fine-grained macro-level hardhats in complex settings (see Table 3). The introduced architecture uses few learning parameters; thus, it is less computationally intensive, which is a critical factor to consider during the model inference process and deployment in the embedded settings. The proposed unified learning model achieved an accuracy of 99.01% and a mean square error of 0.04, which are remarkable in the PPE recognition and classification domain.

The combination of several efficient deep learning architectures yielded a superior, promising predictive and object identification performance that individual learning or constituent models could not accomplish, as observed from the different experiments conducted in this work. The proposed model architectural unification strategy, driven by the ensemble learning, concept minimized the model variance problems through the optimal combination of predictions from the multiple co-learning models, thereby reducing the introduced model sensitivity to specified training algorithms and data. The performance of the proposed unified model simulates an actual real-world situation with minimal variance, reduced overfitting, and enhanced generalization, which led to the emergence of an efficient and rapid processing model. The proposed model is considered to help develop an industrial safety solution to detect the different types of hardhats in the industrial setting to ensure the safety of personnel and other critical infrastructures. The model will be extended to learn diverse safety vests and other safety apparel attributes for a robust and more fine-grain safety PPE classification and macro-level identity recognition in other areas such as disaster scene object, mapping, and classification and recognition.

## Figures and Tables

**Table 1:** The classification and misclassification between the managers’ and site visitors’ test samples.

Custom	T	F	BM1	T	F
Mg	54	6	Mg	53	7
Sv	39	21	Sv	52	8
BM2	T	F	BM3	T	F
Mg	55	5	Mg	55	5
Sv	54	6	Sv	48	12
BM4	T	F	Proposed	T	F
Mg	55	5	Mg	57	3
Sv	55	5	Sv	58	2

**Table 2:** Accuracy and mean matric results from all models.

Model	Test Accuracy	MSE	MSLE	MCC
Custom	0.94	0.23	0.006	0.94
BM1	0.97	0.13	0.004	0.96
BM2	0.98	0.09	0.003	0.97
BM3	0.96	0.14	0.004	0.96
BM4	0.98	0.08	0.002	0.98
Proposed	0.99	0.04	0.001	0.99

**Table 3:** Accuracy and mean matric results from all models.

Method	MAP (Accuracy)
YoloV4	0.859
Centernet2	0.909
Swin-CMR	0.921
YoloV5 with PT	0.922
Proposed	0.99

## Data Availability

The dataset used in this study is available on reasonable request.
